# Supplementary material for: Genomic Assessment of Cancer Susceptibility in the Threatened Catalina Island Fox (Urocyon littoralis catalinae)
Source: Genes (Basel). 2022 Aug 22;13(8):1496. doi: 10.3390/genes13081496 (PMC9408614; doi:10.3390/genes13081496)
Supplement: Supplementary file 1 [file genes-13-01496-s001.zip › Hendricks_CatalinaIslandFox_Supplement_v2.pdf]

Supporting Information

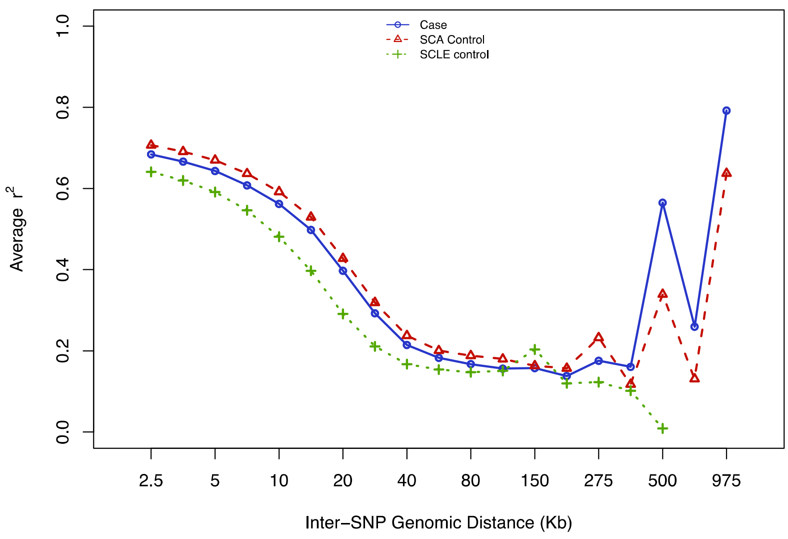

Figure S1. Extent of LD (r^2) as a function of inter-SNP distance (kb) for SCA cases (shown in blue), SCA controls (shown in red), and SCLE controls (shown in green).

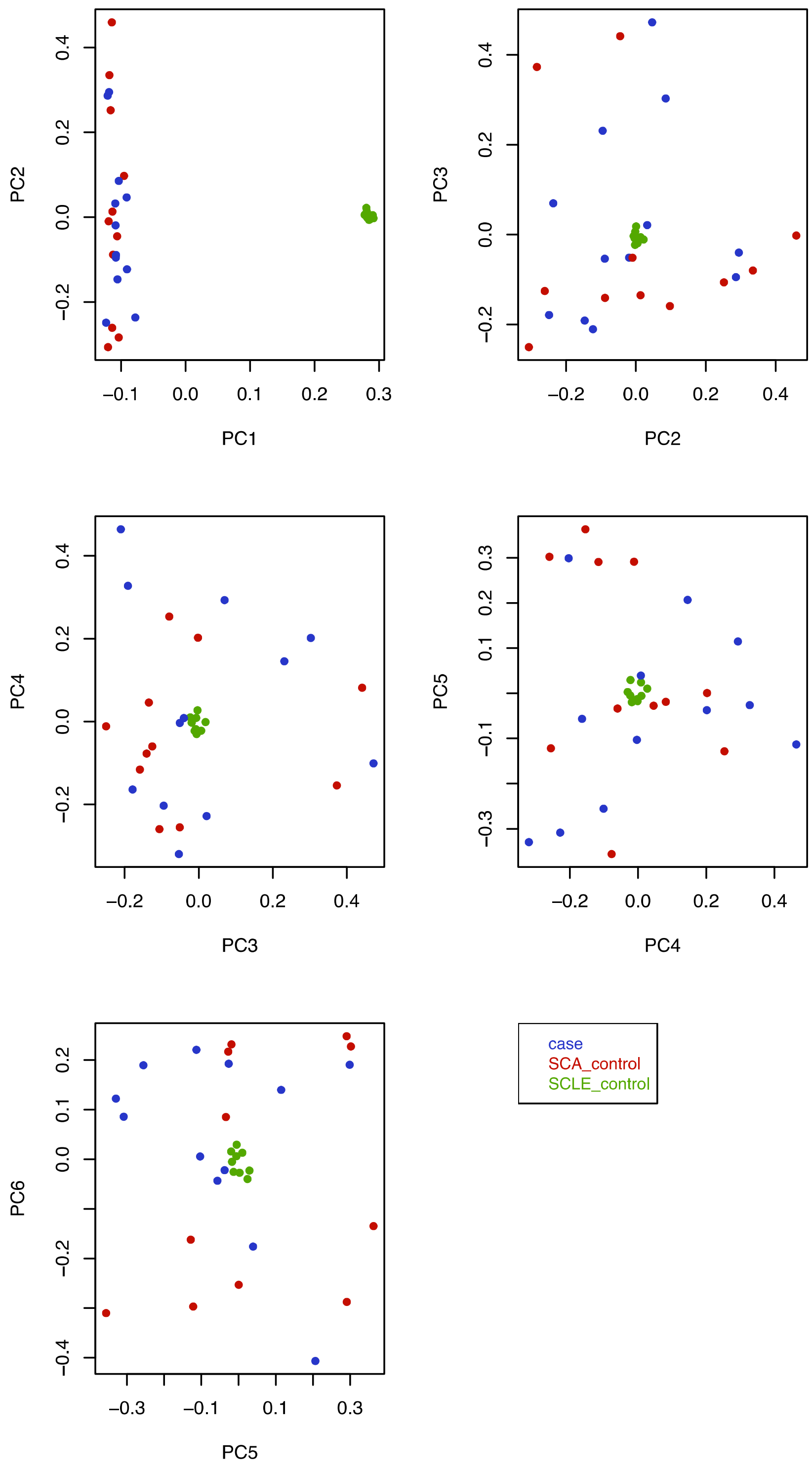

Figure S2. PCA of 32 individuals using 6,600 putatively neutral SNPs. Case individuals are shown in blue; SCA controls in red, and SCLE controls in green.

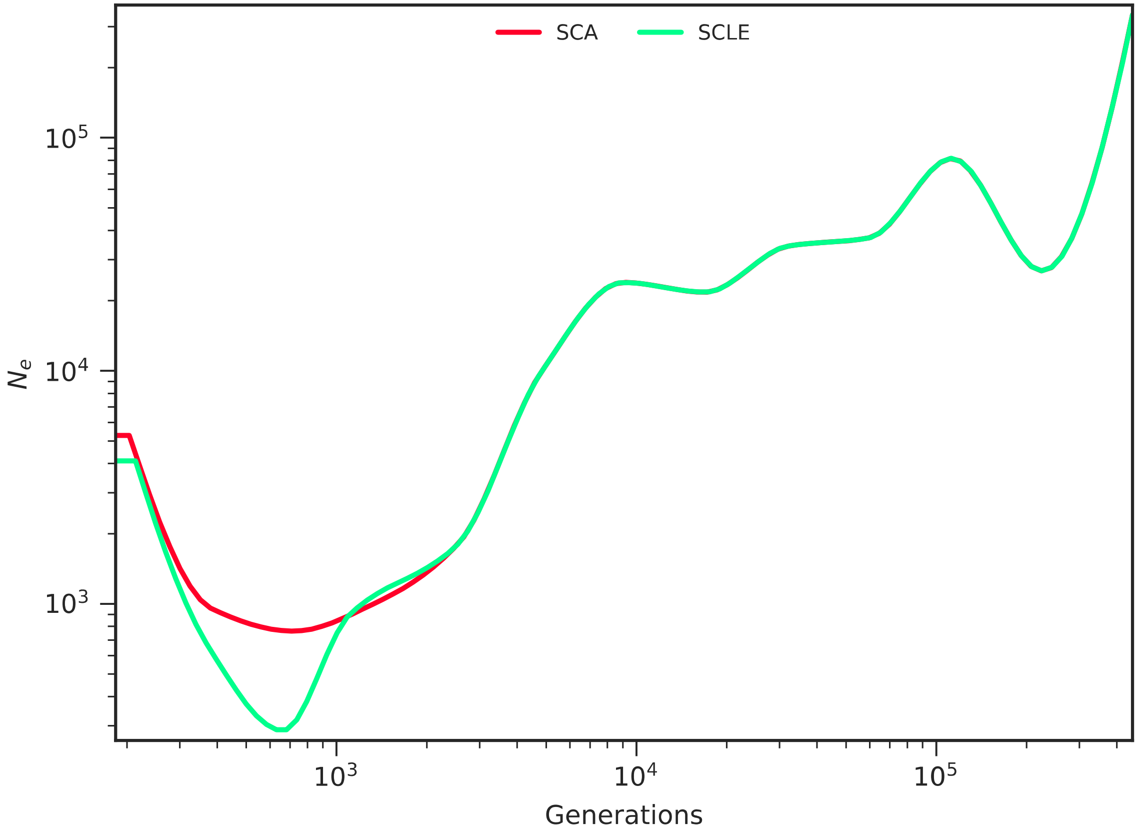

Figure S3. Historical effective population size (N_e) calculated from SNP genotypes within neutral regions. A mutation rate of 1.0 x 10^{-8}/site/generation was assumed (Freedman *et al.* 2014). SCA individuals in red, and SCLE individuals in green.

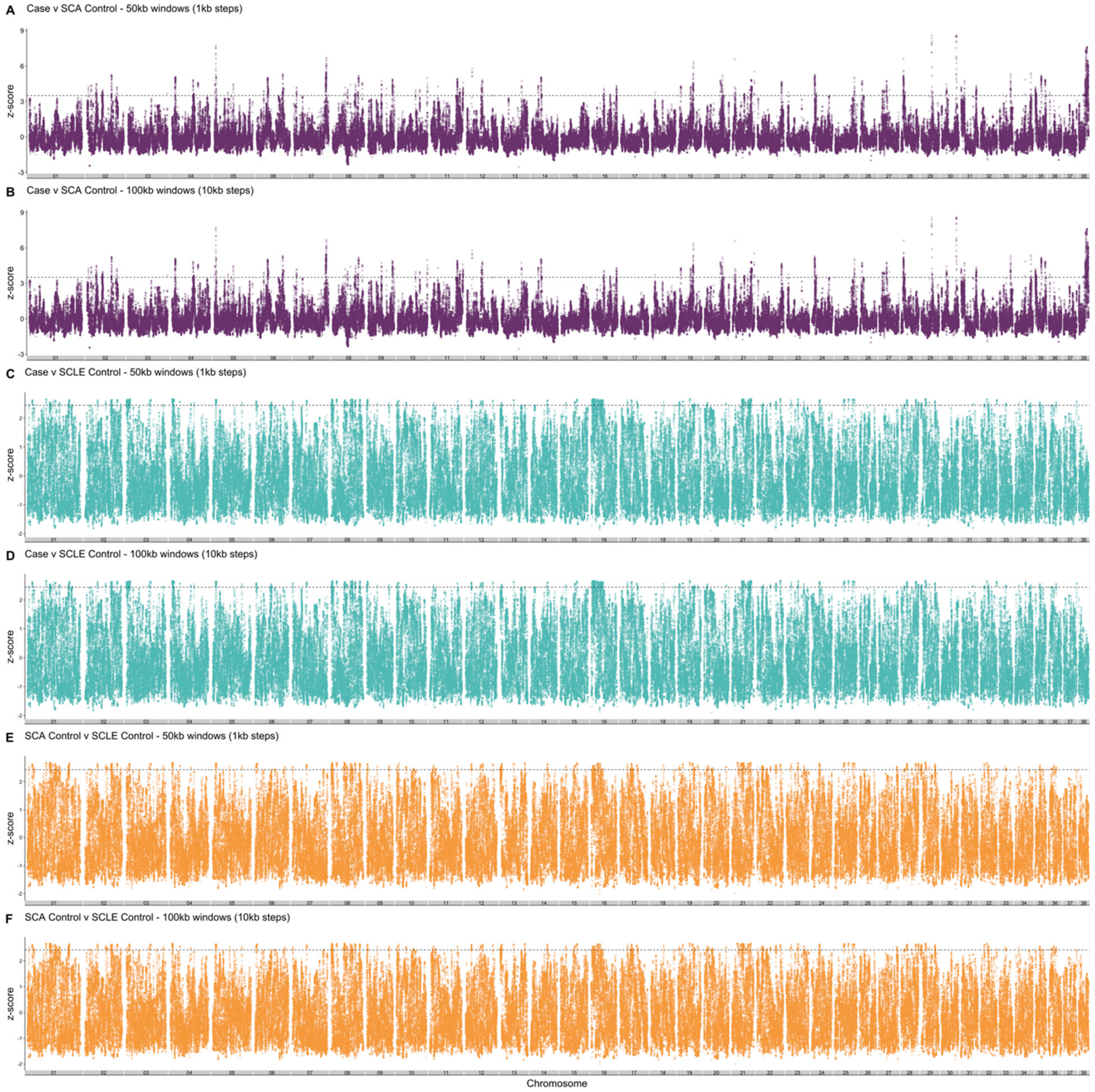

Figure S4. Manhattan plot of F_{ST} values calculated from overlapping 100 kb windows in 10 kb steps using the 35K SNP data set between (A & B) case (n = 12) and SCA control (n = 11) individuals, (C & D) case and SCLE control (n = 9) individuals, (E & F) SCA control and SCLE control individuals. Dashed lines indicated top 1%.

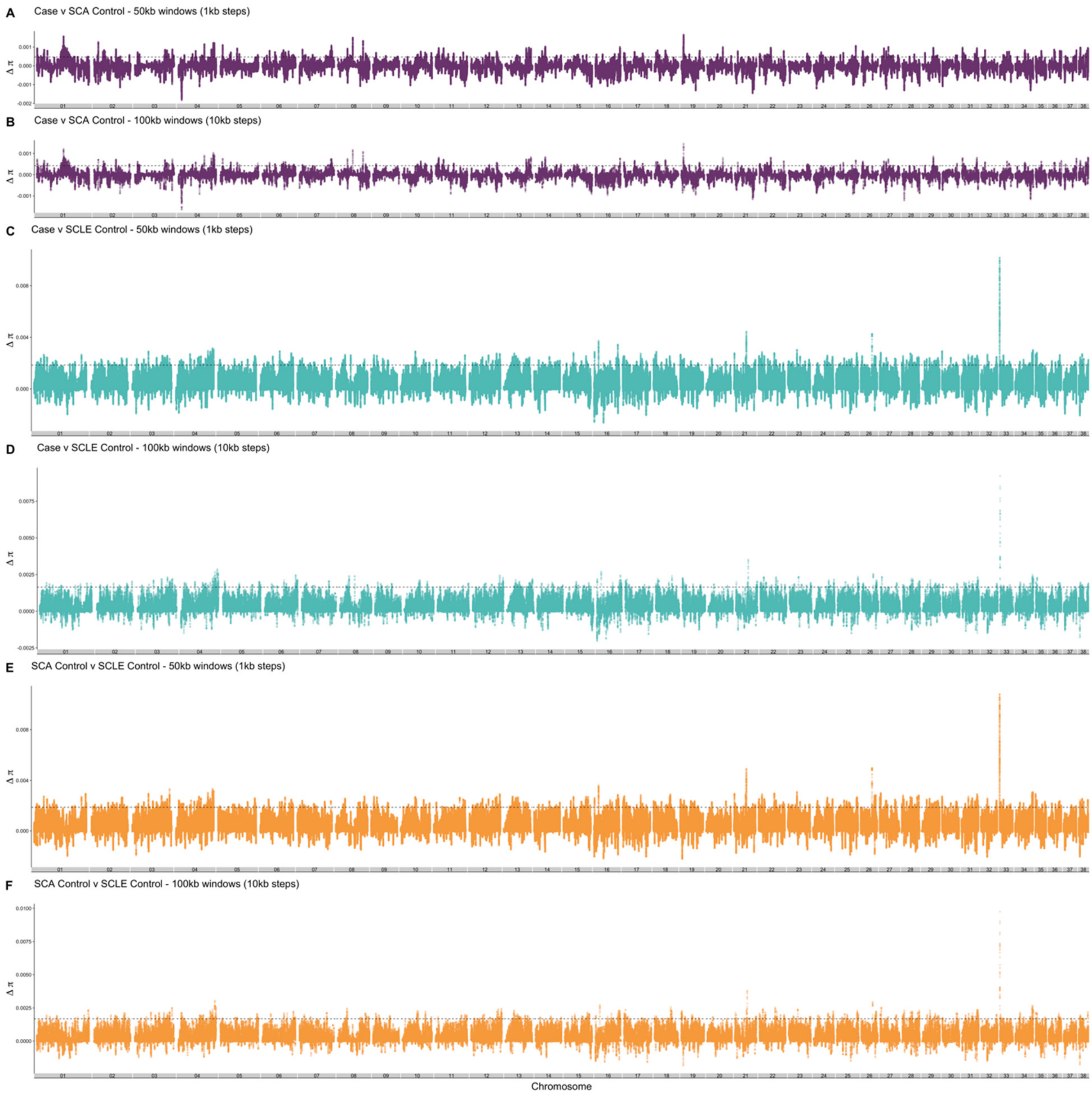

Figure S5. Distribution plots of Δπ values calculated from 50 kb (1 kb steps) and 100 kb (10 kb steps) windows using the 35K SNP data set between (A & B) case (n = 12) and SCA control (n = 11) individuals, (C & D) case and SCLE control (n = 9) individuals, (E & F) SCA control and SCLE control individuals. Dashed lines indicated top 1%.

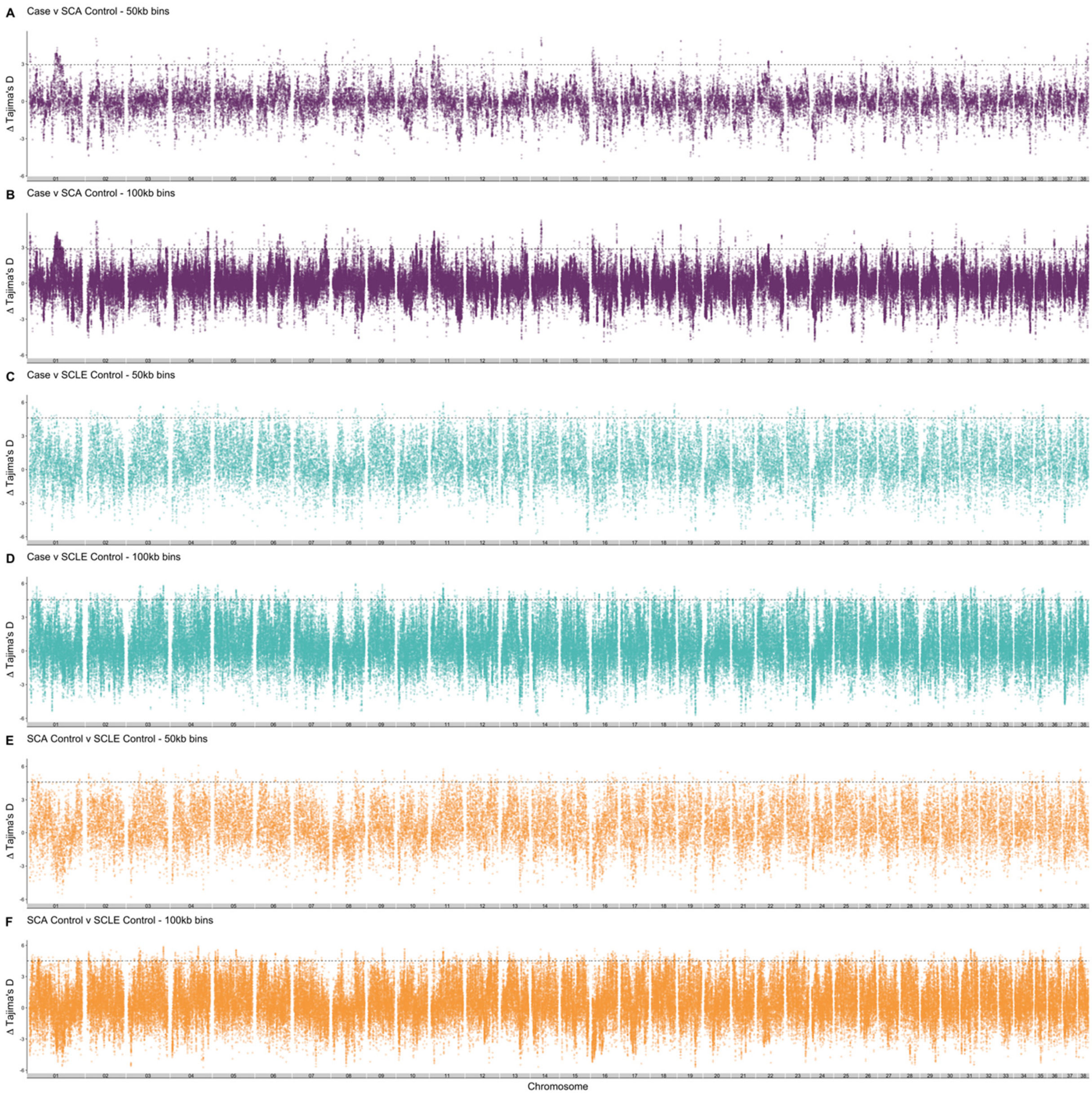

Figure S6. Distribution plots of ΔTajima's D values calculated in 50 kb and 100 kb bins using the 35K SNP data set between (A & B) case and SCA control individuals, (C & D) case and SCLE control individuals, (E & F) SCA control and SCLE control individuals. Dashed lines indicated top 1%.

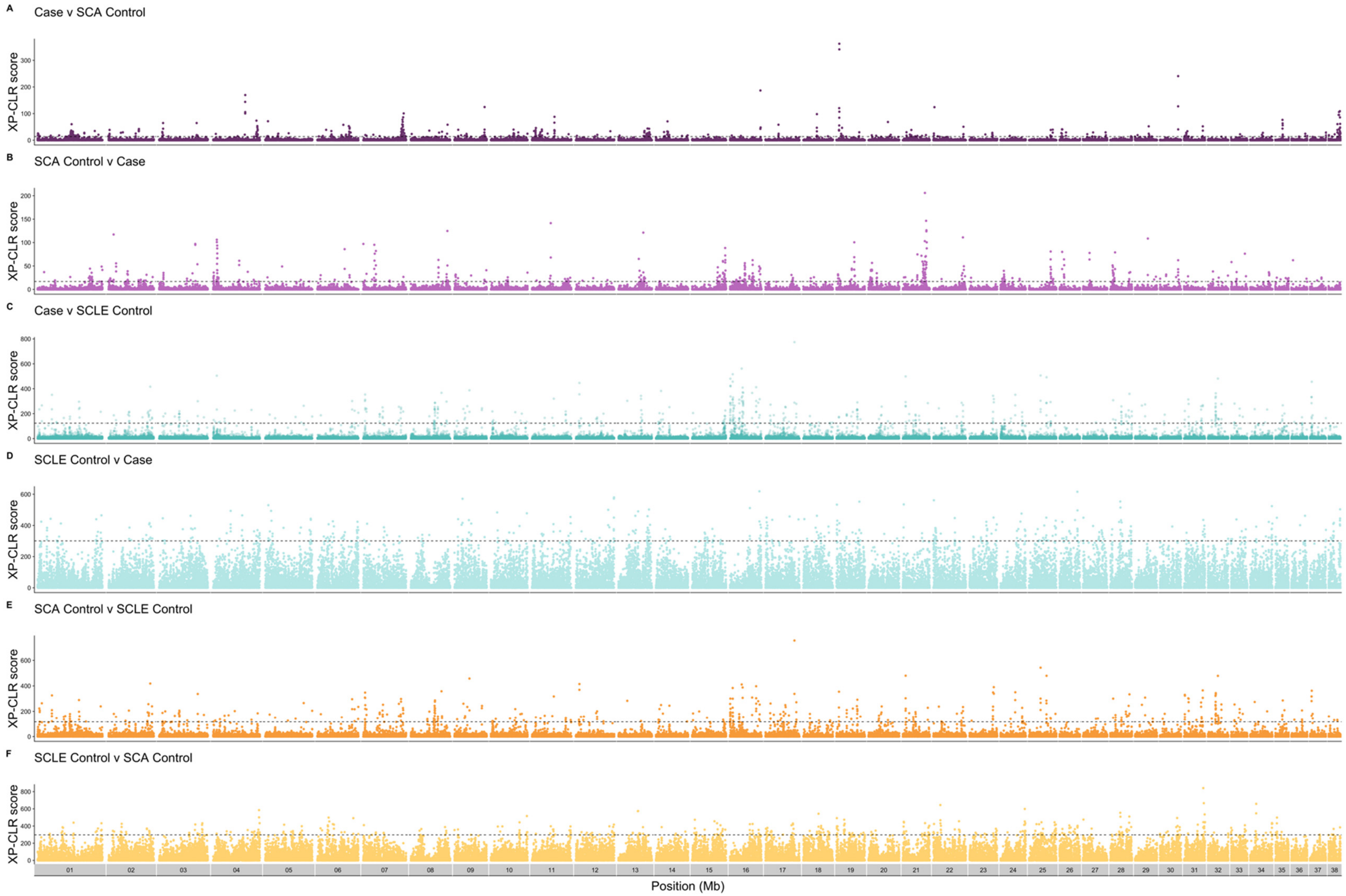

Figure S7. Plot of XP-CLR scores along each autosome in a comparison of all groups. Dashed line indicated top 1%.

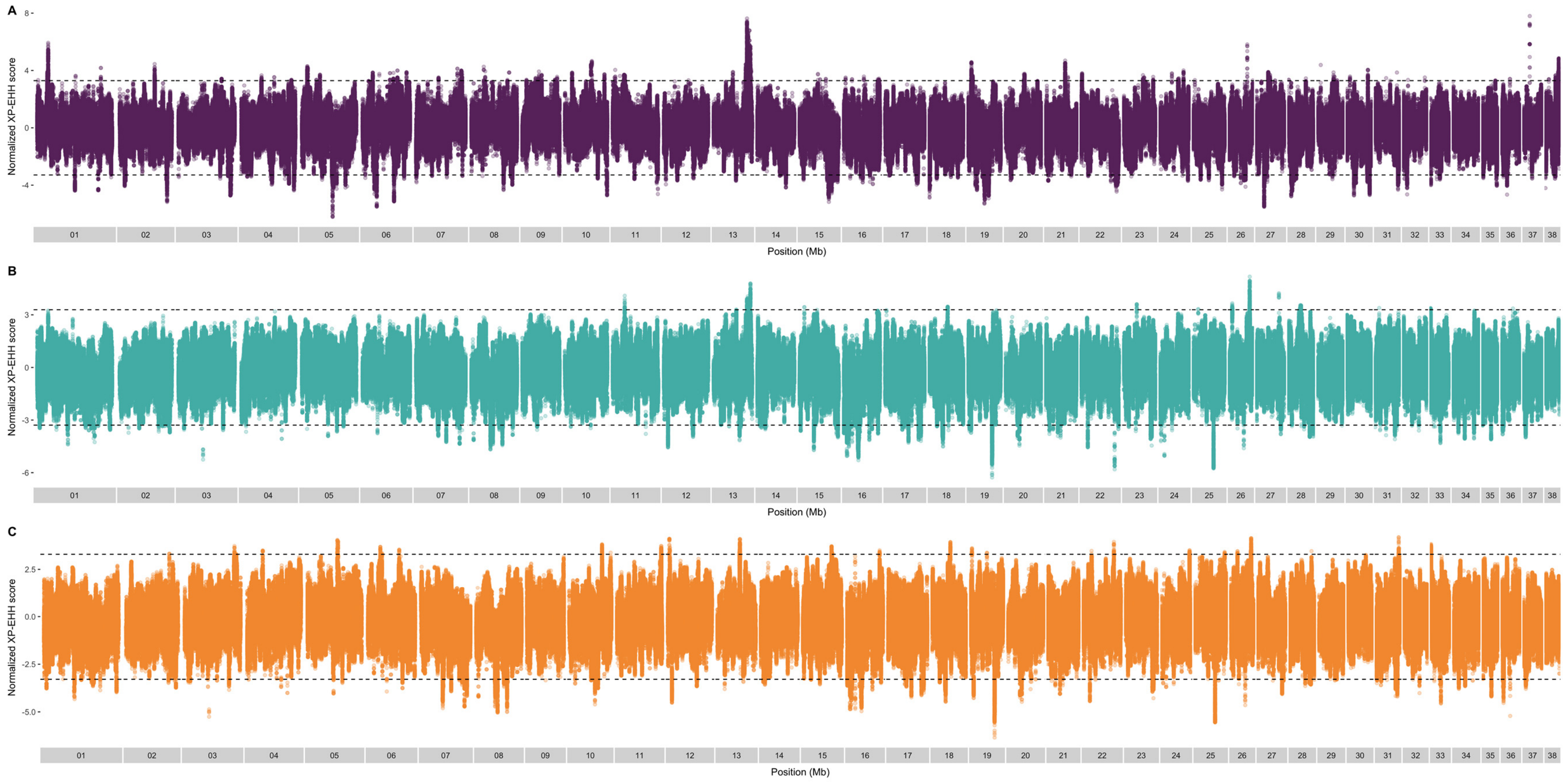

Figure S8. Plot of standardized XP-EHH scores along each autosome in comparisons of (A) case and SCA control individuals, (B) case and SCLE control individuals, (E) SCA control and SCLE control individuals. Dashed line indicates p-value of 0.001 (z-score = ±3.29).

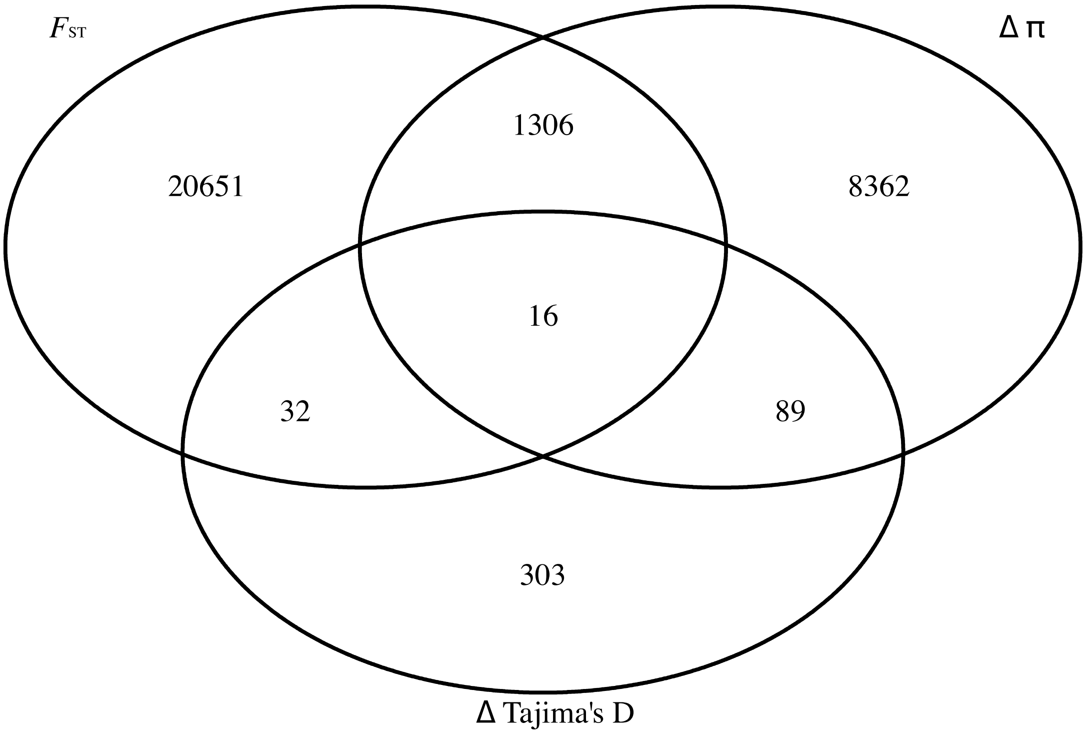

Figure S9. Venn diagram of top 1% of F_{ST}, Δπ, and ΔTajima's D to identify regions under selection.

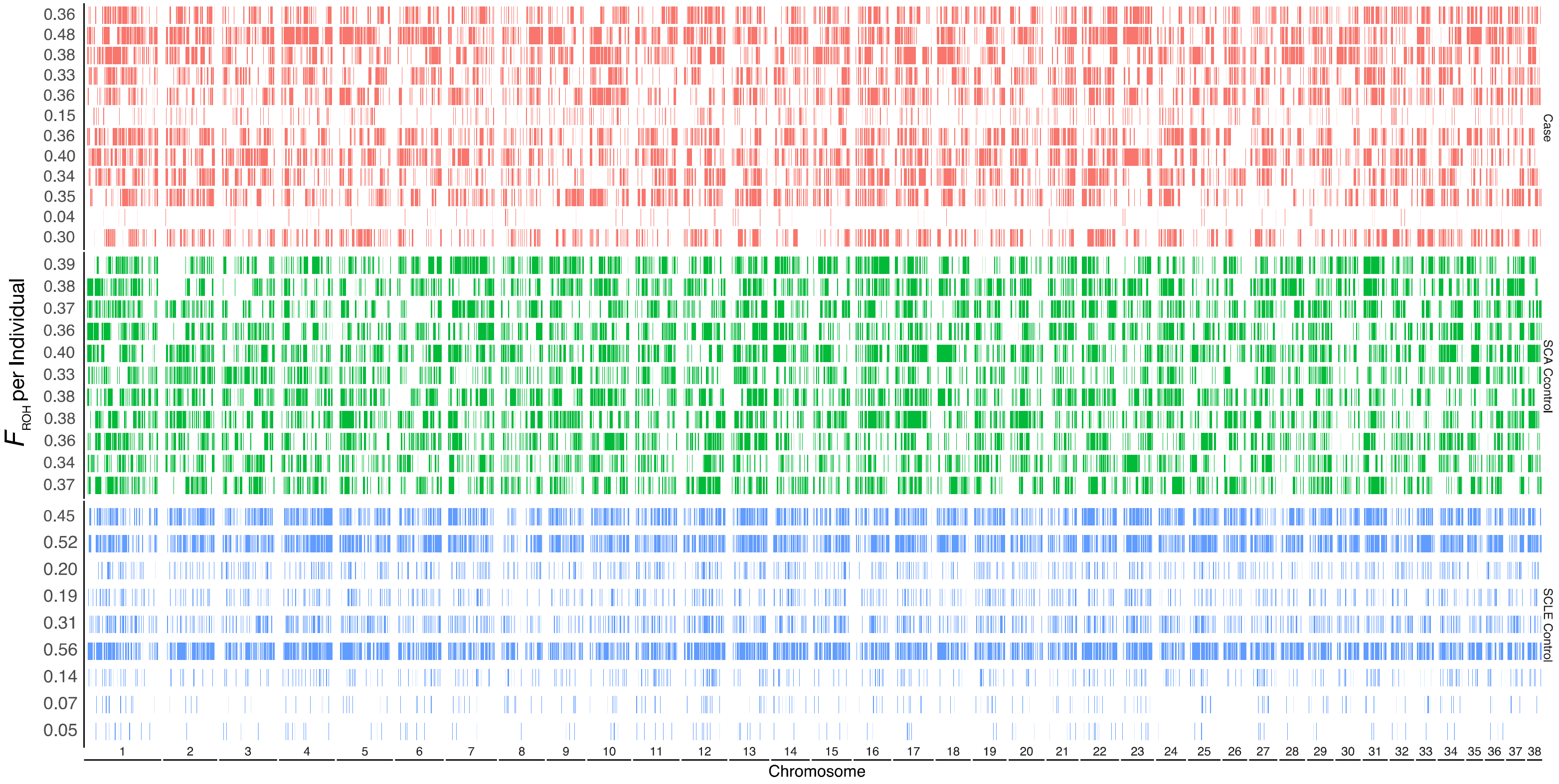

Figure S10. Runs of homozygosity (ROH) for each individual across the genome. The y-axis indicates F_{ROH}, which ranges from 0 to 1, for each individual. The individuals are separated by population with cases shown in salmon, SCA controls in green, and SCLE controls in light blue.

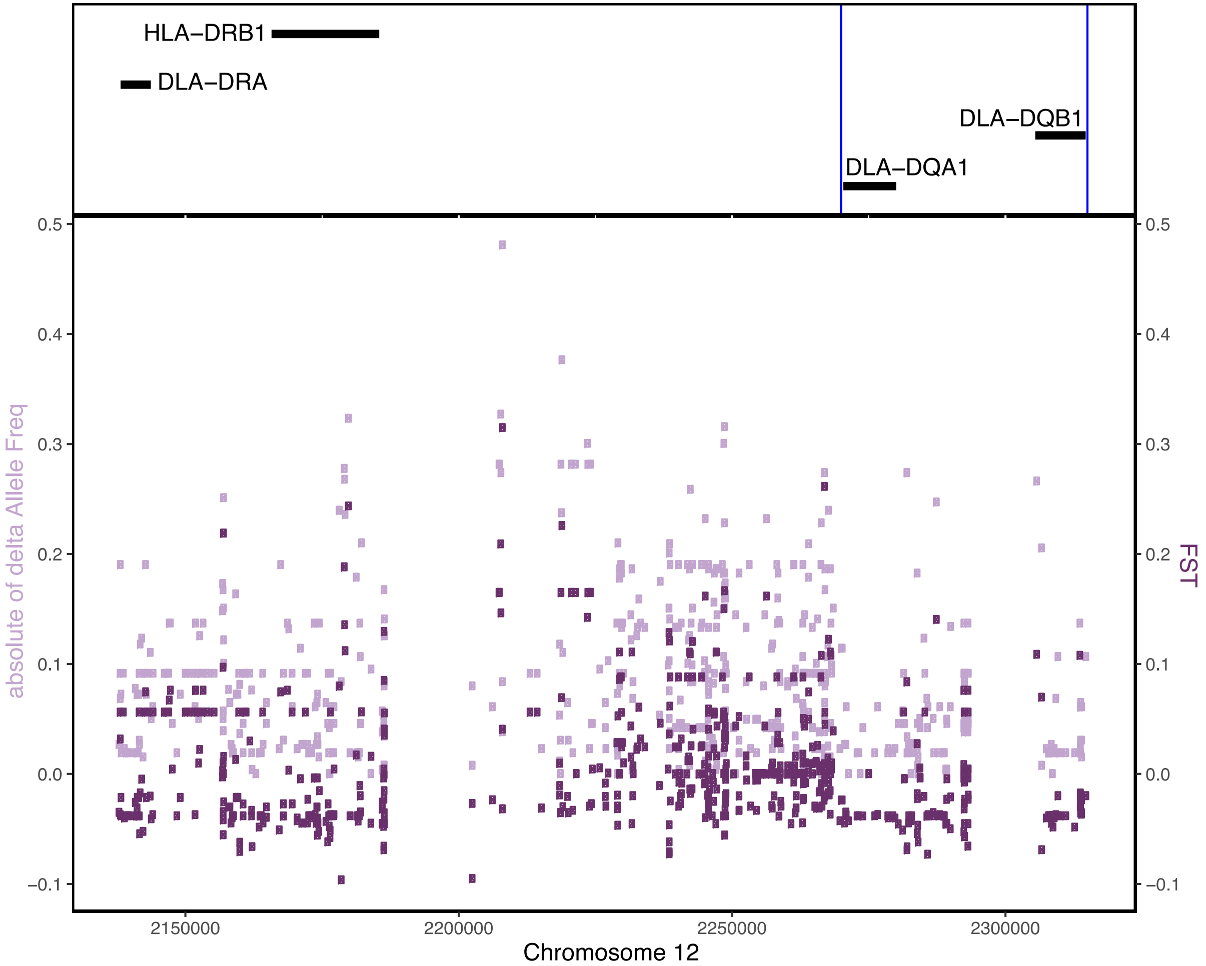

Figure S11. F_{ST} values (dark purple) and absolute values of the difference in allele frequency (light purple) between case and SCA controls within MHC region. Gene annotation show genes as black horizontal bars with TFBS mutations as blue vertical lines.

Supplemental File S1
Supplemental Discussion of Candidate Genes

Candidate genes for cancer susceptibility: Oxidative Stress

The two significant genes involved in superoxide anion generation are Decapping MRNA 1A (*DCP1A*) and C-Reactive Protein (*CRP*). The *DCP1A* gene is part of the PIWI-piRNA pathway and functions to degradation of normal mRNA turnover and nonsense-mediated mRNA as well as TGF-beta signaling pathway. Genetic mutations within this gene have been found to predict survival in melanoma patients (Zhang et al. 2016). The second gene, *CRP* encodes for a protein that is produced mainly in the liver in response to cytokines during infection, trauma, advanced cancer, and chronic inflammatory conditions (Gabay & Kushner 1999). This protein is often measured in order to predict disease such as cardiovascular disease in women (Ridker et al. 2000) and various types of cancer (Heikkilä et al. 2007; Allin et al. 2009). Several studies have shown genetic variants in the *CRP* gene affect blood concentrations of *CRP* and colorectal cancer risk and survival (Erlinger et al. 2004; Nimptsch et al. 2015).

The two significant GO terms both involve the (positive) regulation of superoxide anion generation. Several lines of evidence suggest that an excessive generation of the reactive oxygen species superoxide anion ($O_2^{\cdot -}$) is a key event in cancer development and survival (reviewed in (Lázaro 2007; Kumari et al. 2018). An increase in the cellular production of $O_2^{\cdot -}$ may activate glycolysis, which has been associated with invasion, metastasis, angiogenesis and cell proliferation (Gatenby & Gillies 2004). Simultaneously, $O_2^{\cdot -}$ generation activates Hypoxia-inducible factor 1 (*HIF-1*; Wang et al. 2004a; López-Lázaro 2006). Activation of *HIF-1* can induce apoptosis resistance, invasion, metastasis and angiogenesis (Semenza 2003; Yeo et al. 2004) and may also produce cellular immortalization via activation of telomerase (Nishi et al. 2004).

Furthermore, a high concentration of reactive oxygen species, such as $O_2^{\cdot -}$, has been shown to produce oxidative damage of DNA, causing mutations which eventually lead to cancer (reviewed in Kumari et al. 2018). Therefore, the genes (*DCP1A* and *CRP*) and surrounding intergenic regions may be good candidates for a subsequent fine-mapping study using more individuals to identify potential causal loci underlying cancer risk in the SCA population.

Several candidate genes, including *DCP1A* and *CRP*, interact with phosphate (Soelter & Uhlenbruck 1986; Blumenthal et al. 2009) and potentially are involved in phosphocholine metabolism, a marker for oxidative stress (Frey et al. 2000; Mateos et al. 2008). Some studies have shown that elevated concentrations of phosphate increase cell proliferation and expression of protumorigenic genes (Camalier et al. 2010; 2013). Two of these genes, both members of the Protein Tyrosine Phosphatase (PTP) family, were found with differentiated SNPs between cases and controls. These two genes are Dual Specificity Phosphatase 23 (ENSCAFG00000031934 (*DUSP23*) and Protein tyrosine phosphatase μ (*PTPRM*; also named *PTPμ*). In general, PTPs are susceptible to oxidative stress and loss-of-function mutations involving PTPs are frequently observed in various types of cancers (Wang et al. 2004b; Chen et al. 2006; Flavell et al. 2008; Novellino et al. 2008; Cheung et al. 2008; Chan & Heguy 2009). *PTPRM* is a member of the type IIb subfamily of receptor PTPs (Lamprianou & Harroch 2006) and is involved in cell-cell adhesion (Brady-Kalnay & Tonks 1993). Cleavage, by multiple proteases, of *PTPRM* regulates cell migration in glioblastoma, an aggressive form of brain cancer (Burgoyne et al. 2009; Phillips-Mason et al. 2014). Promotor hypermethylation result in the loss of function of *PTPRM* and has been shown in some acute lymphocytic leukemia (Stevenson et al. 2014) and can lead to colony formation in colon cancer (Sudhir et al. 2015). Additionally, decreased expression of *PTPRM* is associated with poor prognosis in breast cancer (Sun et al. 2012). Atypical dual specificity phosphatases have been implicated in various types of cancer (L & Beeser 2013). VHZ

protein, which is encoded for by *DUSP23*, was overexpressed in breast cancers and various other tumor types (Tang et al. 2010). Further investigation of these two phosphatases will help to increase understanding of the molecular mechanism responsible for its role in promoting cell migration and cell proliferation.

Many of the putative candidate genes have previously been shown to be involved in tumorgenesis. For example, Protein kinase C (*PKC*) is a family of isozymes that play major roles in the control of signaling pathways associated with proliferation, migration, invasion, tumorigenesis, and metastasis (Cooke *et al.* 2017). *PKC* represents one of the most extensively studied kinases, with >60,000 citations in PubMed and >10,000 citations associated with cancer (Cooke *et al.* 2017). *PKCδ* mainly functions as an anti-proliferative kinase that negatively regulates cell cycle progression (Griner & Kazanietz 2007). Furthermore, overexpression of *PKCδ* in the epidermis of transgenic mice protects against tumorigenesis when exposed to the tumor promoting phorbol esters (Reddig *et al.* 1999). Contrary to this, *PKCδ* has tumor promoting activity in mammary gland and pancreatic tumorigenesis (Mauro *et al.* 2010; Allen-Petersen *et al.* 2014).

Reticulon 1 (*RTN1*) proteins are members of highly conserved reticulons, which are localized in the endoplasmic reticulum (ER). Reticulons show pro-apoptotic activity via the induction of ER stress (Kuang *et al.* 2005; Di Sano *et al.* 2007). Reduced expression of *RTN1* in colon adenocarcinomas (Lemire *et al.* 2015) and highly expressed in small cell and non-small cell lung cancers (van de Velde *et al.* 1994; Senden *et al.* 1996; 1997a; b). *DPP10* may play a role in disease progression of colorectal cancer and loss of *DPP10* expression in primary CRC is significantly associated with poor survival outcomes (Park *et al.* 2013). Another gene, Cilia And Flagella Associated Protein 45 (*CFAP45*; also known as Coiled-Coil Domain Containing 19) is highly associated with both pharynx, nasopharyngeal, and lung carcinoma in humans (Liu *et al.* 2011) (Wang *et al.* 2018). This gene has been found to regulate miR-184 and thereby suppress cell

proliferation, invasion and migration (Liu *et al.* 2014). Decreased expression of *CCDC19* is correlated with poor prognosis in lung cancer patients (Wang *et al.* 2018). With SNPs within the TFBS's and strong evidence for selection across these regions, these genes are likely involved in tumor suppression/promotion in SCA foxes.

Immunological surveillance hypothesis states that in large, long-lived animals, heritable genetic changes are common in somatic cells and represent progression to malignancy. Further, there must be a mechanism, likely immunological in character, for eliminating potentially dangerous mutant cells. In the case of SCA and ceruminous gland carcinoma, two genes, *FCRL6* and ENSCAFG00000011795 (Ortholog of *SLAMF8*), were both in regions of the genome highly differentiated between cases and controls, have immunological functions, and disruption in these genes have been found to lead to tumorigenesis. For example, *FCRL6* distinguishes mature cytotoxic lymphocytes and has been found to be upregulated in patients with B-cell chronic lymphocytic leukemia (Schreeder *et al.* 2008). Slam family member antigens are lymphocyte activation proteins involved in the pathogenesis of immunological disorders, and may also contribute to the activation of cancer cells (Furukawa *et al.* 2010). Exploring expression of these genes may be informative to better understand cancer susceptibility in SCA foxes.

Long non-coding RNAs (>200 nts; lncRNAs) play critical roles in gene transcription, translation, and chromatin modification. Most large intergenic noncoding RNAs (lincRNAs), a subclass lncRNAs, are enriched in evolutionarily conserved sequences and therefore likely functional (Khalil et al. 2009); yet are rarely functionally annotated (Quek et al. 2015). lncRNAs compete with proteins in terms of their diversity and regulatory potential through a wide range of mechanisms such as chromatin and methylation modification and activation, direct effect on stability of protein and

protein complexes, or by acting as a sponge for miRNA inhibition (Quinn & Chang 2016; Bartonicek et al. 2016). lncRNAs can modify the phosphorylation state of proteins by masking phosphorylation motifs (Liu et al. 2015). lncRNAs as regulatory molecules have been implicated in the majority of these hallmarks of cancer (reviewed in (Gutschner & Diederichs 2012; Bartonicek et al. 2016)). The hallmarks of cancer or the six properties required for cell transformation in tumorigenesis are self-sustained growth signaling, insensitivity to growth inhibition, apoptosis avoidance, uncontrolled proliferation, angiogenesis and metastasis (Hanahan & Weinberg 2000; 2011). It may be plausible that the three lincRNA's (ENSCAFG00000039008, ENSCAFG00000039447, and ENSCAFG00000039265) we found with downstream and intronic SNPs may have a role in cancer susceptibility in SCA foxes. Additionally, ENSCAFG00000030255 (DLX6 antisense RNA 1; *DLX6-AS1*) is a developmentally-regulated long non-coding RNA that also contained a highly differentiated SNP between cases and controls. High *DLX6-AS1* expression was noticed in lung adenocarcinoma and associated with histological differentiation and TNM Classification of Malignant Tumors stage (Li et al. 2015). *DLX6-AS1* was also up-regulated in hepatocellular carcinoma tissue and correlated with clinical prognosis (Li et al. 2017). Future studies of these lncRNA's should be explored to better elucidate the pathways involved in cancer susceptibility in SCA foxes.

References:

1. Allen-Petersen BL, Carter CJ, Ohm AM, Reyland ME (2014) Protein kinase Cδ is required for ErbB2-driven mammary gland tumorigenesis and negatively correlates with prognosis in human breast cancer. *Oncogene*, **33**, 1306–1315.
2. Bartonicek N, Maag JLV, Dinger ME (2016) Long noncoding RNAs in cancer: mechanisms of action and technological advancements. *Molecular Cancer*, **15**, 43.
3. Cooke M, Magimaidas A, Casado-Medrano V, Kazanietz MG (2017) Protein kinase C in cancer: The top five unanswered questions. *Molecular carcinogenesis*, **56**, 1531–1542.
4. Di Sano F, Fazi B, Tufi R, Nardacci R, Piacentini M (2007) Reticulon-1C acts as a molecular switch between endoplasmic reticulum stress and genotoxic cell death pathway in human neuroblastoma cells. *Journal of neurochemistry*, **102**, 345–353.
5. Freedman AH, Gronau I, Schweizer RM *et al.* (2014) Genome sequencing highlights the dynamic early history of dogs. *PLoS Genetics*, **10**, e1004016–12.

6. Furukawa H, Tohma S, Kitazawa H *et al.* (2010) Role of SLAM-associated protein in the pathogenesis of autoimmune diseases and immunological disorders. *Archivum immunologiae et therapiae experimentalis*, **58**, 37–44.
7. Griner EM, Kazanietz MG (2007) Protein kinase C and other diacylglycerol effectors in cancer. *Nature Reviews Cancer*, **7**, 281–294.
8. Gutschner T, Diederichs S (2012) The hallmarks of cancer. *RNA Biology*, **9**, 703–719.
9. Hanahan D, Weinberg RA (2000) The hallmarks of cancer. *Cell*, **100**, 57–70.
10. Hanahan D, Weinberg RA (2011) Hallmarks of cancer: the next generation. *Cell*, **144**, 646–674.
11. Khalil AM, Guttman M, Huarte M *et al.* (2009) Many human large intergenic noncoding RNAs associate with chromatin-modifying complexes and affect gene expression. *Proceedings of the National Academy of Sciences*, **106**, 11667–11672.
12. Kuang E, Wan Q, Li X *et al.* (2005) ER Ca2+ depletion triggers apoptotic signals for endoplasmic reticulum (ER) overload response induced by overexpressed reticulon 3 (RTN3/HAP). *Journal of Cellular Physiology*, **204**, 549–559.
13. Lemire M, Qu C, Loo LWM *et al.* (2015) A genome-wide association study for colorectal cancer identifies a risk locus in 14q23.1. *Human Genetics*, **134**, 1249–1262.
14. Li J, Li P, Zhao W *et al.* (2015) Expression of long non-coding RNA DLX6-AS1 in lung adenocarcinoma. *Cancer Cell International*, **15**, 48.
15. Li L, Hou A, Gao X *et al.* (2017) Lentivirus-mediated miR-23a overexpression induces trophoblast cell apoptosis through inhibiting X-linked inhibitor of apoptosis. *Biomedicine & pharmacotherapy*, **94**, 412–417.
16. Liu B, Sun L, Liu Q *et al.* (2015) A cytoplasmic NF-κB interacting long noncoding RNA blocks IκB phosphorylation and suppresses breast cancer metastasis. *Cancer cell*, **27**, 370–381.
17. Liu Z, Li X, He X *et al.* (2011) Decreased expression of updated NESG1 in nasopharyngeal carcinoma: Its potential role and preliminarily functional mechanism. *International Journal of Cancer*, **128**, 2562–2571.
18. Liu Z, Mai C, Yang H *et al.* (2014) Candidate tumour suppressor CCDC19 regulates miR-184 direct targeting of C-Myc thereby suppressing cell growth in non-small cell lung cancers. *Journal of Cellular and Molecular Medicine*, **18**, 1667–1679.
19. Mauro LV, Grossoni VC, Urtreger AJ *et al.* (2010) PKC Delta (PKCδ) Promotes Tumoral Progression of Human Ductal Pancreatic Cancer. *Pancreas*, **39**, e31–e41.
20. Park HS, Yeo HY, Chang HJ *et al.* (2013) Dipeptidyl peptidase 10, a novel prognostic marker in colorectal cancer. *Yonsei Medical Journal*, **54**, 1362–1369.
21. Quek XC, Thomson DW, Maag JLV *et al.* (2015) lncRNAdb v2.0: expanding the reference database for functional long noncoding RNAs. *Nucleic Acids Research*, **43**, D168–73.
22. Quinn JJ, Chang HY (2016) Unique features of long non-coding RNA biogenesis and function. *Nature Reviews Genetics*, **17**, 47–62.
23. Reddig PJ, Dreckschimdt NE, Ahrens H *et al.* (1999) Transgenic Mice Overexpressing Protein Kinase Cδ in the Epidermis Are Resistant to Skin Tumor Promotion by 12-O-Tetradecanoylphorbol-13-acetate. *Cancer Research*, **59**, 5710–5718.
24. Schreeder DM, Pan J, Li FJ, Vivier E, Davis RS (2008) FCRL6 distinguishes mature cytotoxic lymphocytes and is upregulated in patients with B-cell chronic lymphocytic leukemia. *European Journal of Immunology*, **38**, 3159–3166.
25. Senden NHM, Timmer EDJ, Bruïne A de *et al.* (1997a) A Comparison of NSP-Reticulons with Conventioal Neuroendocrine Markers in Immunophenotyping of Lung Cancer. *The Journal of Pathology*, **182**, 13–21.
26. Senden NH, Timmer ED, Boers JE *et al.* (1996) Neuroendocrine-specific protein C (NSP-C): subcellular localization and differential expression in relation to NSP-A. *European journal of cell biology*, **69**, 197–213.
27. Senden N, Linnoila I, Timmer E *et al.* (1997b) Neuroendocrine-specific protein (NSP)-reticulons as independent markers for non-small cell lung cancer with neuroendocrine differentiation. *Histochemistry and Cell Biology*, **108**, 155–165.
28. van de Velde HJ, Senden NH, Roskams TA *et al.* (1994) NSP-encoded reticulons are neuroendocrine markers of a novel category in human lung cancer diagnosis. *Cancer Research*, **54**, 4769–4776.

29. Wang Y, Liu Z, Luo R, X Y (2018) Decreased CCDC19 is correlated with unfavorable outcome in lung squamous cell carcinoma. *International Journal of Clinical and Experimental Pathology*, **11**, 802–807.
